# Aptamer: A potential oligonucleotide nanomedicine in the diagnosis and treatment of hepatocellular carcinoma

**DOI:** 10.18632/oncotarget.23359

**Published:** 2017-12-16

**Authors:** Rusdina Bte Ladju, Devis Pascut, Muhammad Nasrum Massi, Claudio Tiribelli, Caecilia H.C. Sukowati

**Affiliations:** ^1^ Fondazione Italiana Fegato, AREA Science Park Basovizza, Trieste, Italy; ^2^ Faculty of Medicine, Hasanuddin University, Makassar, Indonesia

**Keywords:** hepatocellular carcinoma, aptamer, oligonucleotide nanomedicine, future therapy

## Abstract

Hepatocellular carcinoma (HCC) is one of the most common cancers with a high mortality rate. Late diagnosis and poor prognosis are still a major drawback since curative therapies such as liver resection and liver transplantation are effective only for an early stage HCC. Development of novel molecular targeting therapies against HCC may provide new options that will improve the efficiency of the diagnosis and the success of the therapy, thus ameliorating the life expectancy of the patients. The aptamer is an oligonucleotide nanomedicine that has high binding affinity and specificity to small and large target molecules in the intracellular and extracellular environment with agonist or antagonist function. Currently, several aptamers for diagnostic and therapeutic purposes are under development to recognize different molecules of HCC. In *in vitro* models, the aptamer has been shown to be able to reduce the growth of HCC cells and increase the sensitivity to conventional chemotherapies. In *in vivo* mouse models, aptamer could induce cell apoptosis with antitumor activity. Overall data had shown that aptamer has limited toxicity and might be safe in clinical application. This review summarizes recent information of aptamer as a potential oligonucleotide nanomedicine tool, in diagnostics, targeted therapy, and as drug delivery nano-vehicles.

## HEPATOCELLULAR CARCINOMA: CURRENT TREATMENTS AND OBSTACLES

Hepatocellular carcinoma (HCC) is one of most common cancers and the second leading cause of cancer-related death worldwide [1]. The incidence of HCC is expected to increase in the future, especially in America and northern and central Europe where diabetes, obesity, and alcohol abuse represent the major risk factors [2–4].

The success of HCC treatment primarily depends on the time of diagnosis. Early diagnosis is crucial for a favorable prognosis since curative therapies options, such as local radiofrequency ablation and surgical intervention (liver transplantation and liver resection), have a much higher efficacy in the very early and early-stage HCC as compared to later stages [4, 5]. Liver transplantation can be the best treatment for HCC with low risk of recurrence. However, due to the disparity of liver donor resources and the increasing number of patients, it is suggested as a second line treatment only in case of relapse or liver failure after liver resection and ablation therapy [6]. Patients in later stages HCC (intermediate and advanced), can receive palliative treatments such as chemoembolization and kinase inhibitors, while for patients in the terminal stage can only receive best supportive care [7].

Nevertheless, tumor recurrence after percutaneous ablation or liver resection treatment can be a problem also in the early stages HCC. The probability of 5 years HCC recurrence is around 80% after liver resection [8] and 62% after liver ablation [9]. Furthermore, palliative treatments for intermediate and advanced stages often have an unfavorable outcome due either to drug side effects or drug resistance. A recent study done by Njei *et al.* showed that only 46.2% of HCC cases are diagnosed at an early stage where most of the cases do not receive curative therapy [10].

Based on this evidence, HCC treatment options are still hampered by many obstacles. Therefore, the development of early diagnostic tools and new therapeutic approaches will be crucial to improving survival rate and life quality of the patient.

## APTAMER: OLIGONUCLEOTIDES NANOMEDICINE

The use of antibodies has been extensively studied both in research and in clinical application [11]. Although they have the ability to selectively recognize and bind to various biological molecules, the clinical application is still limited by the high immunogenicity, high production cost, and low stability [12, 13].

In recent years, nanomedicine technology represents a promising bench-to-bedside strategy in medicine. The oligonucleotide nanomedicine has been widely studied starting from anti-sense oligonucleotides, aptamers, to small-interference RNA (siRNA). Oligonucleotide nanomedicine has been demonstrated to be a powerful tool both for cancer diagnostic and for cancer therapy. In HCC, oligonucleotide nanomedicine therapy is predicted to achieve a better result than antibody-based therapy due to the non-effective treatment of the tested drug, codrituzumab (antibody-based therapy) against HCC [14, 15].

In January 2017, after the evaluation of strictly controlled trials, the Food and Drug Administration approved the application of six oligonucleotides for therapy [16]. This breakthrough is a very promising prospect for various oligonucleotides nanomedicine, including for the aptamer.

**Figure 1:**
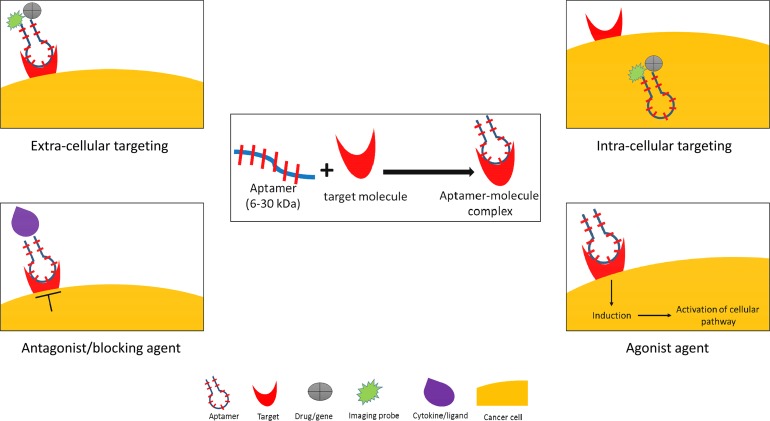
Aptamer applications in cancer medicine Oligonucleotide aptamer can function as both extra- and intra-cellular targeting molecule, and as antagonist and agonist activating molecule. By using conjugation with imaging probe, drug, or gene-therapy, aptamer can be used as a nano-delivery agent in a direct or indirect system.

Aptamers are single-stranded RNA or DNA oligonucleotides with low molecular weight (6-30 kDa) that specifically and efficiently bind to a target molecule [17, 18]. This characteristic makes them suitable for a targeted therapy because of their ability to reach the core of the cancer cells and to internalize through endosomal pathway. Aptamers have a flexible configuration that recognizes and binds to the related target in a specific and high binding affinity *via* an adaptive recognition manner [19]. The aptamer-target complex has very low dissociation constants ranging from picomolar to nanomolar due to the specific hydrogen bonding [20–22]. The aptamers have a unique niche compared to other oligonucleotides. They can be developed to bind an intracellular or extracellular target, and they can be functioned as an agonist or antagonist (Figure 1).

The principle of the aptamer molecular binding is based on its capability to spontaneously fold into a unique three-dimensional (3D) structure without the involvement of covalent bonds [19, 23]. As expected, the effect of aptamer-target interaction depends on the molecular function and cellular localization of the target molecule. In some cases, the aptamer-target complex can block the interaction between a ligand and its receptors that subsequently stimulates the cellular response [24] as for example, an immune response against viral infection [25, 26]. Some aptamers also have agonist-like activities that can enhance and induce protein synthesis [27, 28].

Aptamers are also known as “chemical antibodies” since they can bind specifically to target molecules either in the intracellular or extracellular environment [20, 29]. Aptamers have high versatility in targeting different molecules of different nature, size, and complexity, ranging from ions to whole cell, antibiotic, protein, bacteria, and virus [30]. However, compared to conventional antibodies, aptamers exhibit significant advantages. Aptamer commonly sustains its specificity and sensitivity by binding their ligands *via* adaptive recognition involving conformational alteration and molecular shape complementary [31]. They are stable at room temperature and in non-physiological conditions, non-immunogenic, non-toxic, and suitable for long-term repeated administration [32–34]. They also have high bio-distribution in biological fluids [35, 36] and high capacity to penetrate and remain in the tumor site [37]. The low-cost and well-standardized chemical synthesis, which is 1000 times cheaper than the antibodies production [38, 39], are also important aspects.

The aptamer can be generated by using Systematic Evolution of Ligands by Exponential Enrichment (SELEX), first described by Tuerk and Ellington [40, 41]. SELEX method is based on chemical process consisting of selection (binding phase, partitioning phase, and elution phase), amplification, and conditioning [42]. In the binding phase, a random nucleic acid library is incubated with the target molecule, followed by the partitioning phase that separates target-bound oligos from the remaining unbound library. The bound oligos are then eluted and are amplified to generate an enriched pool of aptamers candidates. The process can be repeated for 8-20 cycles to obtain candidates with the highest affinity to the target molecule. Finally, the sequences of chosen aptamers are characterized by sequencing [43].

Since its launch in 1990, conventional SELEX has been progressively modified to improve aptamer specificity and affinity, to simplify the process and to increase time and cost efficiency [44–46]. The modifications in the SELEX method depend on the purpose and target molecules. For example, *in vivo* SELEX, cell-SELEX, one-round SELEX, *in silico* SELEX, capillary electrophoresis-SELEX, magnetic bead-based SELEX, and high-throughput sequencing-SELEX are several novel SELEX technologies that are already well established [47]. Several SELEX methods are listed in Table 1.

**Table 1:** Modified SELEX methods

SELEX methods	Principle	Ref
*In vivo*	Localizing target molecules inside a living cell	[90]
Cell-based	Targeting the whole live cell	[91]
One-round step	One selection rounds of aptamer generation	[92]
*In silico*	Computational docking technology	[93]
Capillary electrophoresis	Electrophoretic mobility based separation	[94]
Magnetic bead-based	Magnetic beads immobilization	[95]
High-throughput sequencing-based	High-throughput sequencing and bioinformatics analysis	[96]
Ligand-guided selection	Using specific antibody to compete with the target molecule	[97]
Isogenic cell	Isogenic cell line application in counter selection step	[98]
Quantitative parallel aptamer selection system	Combination of microfluidic, next generation sequencing, and *in situ*-synthesized arrays	[99]

## DEVELOPMENT OF APTAMER AGAINST HCC

As mentioned above, the aptamer is a potent tool in basic and clinical biomedicine. Until now, numerous aptamers for different biomedical applications as biosensor and imaging nanoparticle for diagnostic [48–50], drug delivery agent [51–53], and theranostic (therapy and diagnostic) [54–56] had been discovered. Since Pegaptanib, an aptamer targeting vascular endothelial growth factor (VEGF), had been approved for age-related macular degeneration treatment [57], several aptamers had been shown to be a promising tool in clinical applications. Aptamer AS1411 (for acute myeloid leukemia and renal cell carcinoma) and NOX-A12 (for chronic lymphocytic leukemia and refractory multiple myeloma) are currently used in clinical trials [58, 59]. AS1411 was shown to selectively recognize cancer cells *in vivo* without any major side effects and toxicity [59]. Meanwhile, several aptamers are still under preclinical trials.

The increasing number of cases and poor prognosis of HCC highlight the need for a significant, appropriate, and efficient management of the disease. The screening and verification of potential aptamers as molecular probes against HCC will be needed to discover novel biomarkers in diagnostic and therapeutic implications [60]. An aptamer against Lipocalin-2 (a 24kDa secretory glycoprotein) had been proposed as an effective biomarker in HCC that may improve the diagnosis [61]. It had been reported that aptamers could also be used to monitor the progression of HCC by detecting metastatic cells [62] and circulating tumor cells [63]. The current evidence on the development of aptamers for HCC diagnosis, targeted therapy, and theranostic approach are listed in Table 2.

**Table 2:** Aptamers development against HCC

Aptamer (Oligotype)	Target	Principle	Function	Ref.
LNC_2__apta_2&4_, (DNA)	Lipocalin-2	Sandwich-based assay	Detection	[61]
Aptamer C-2 (DNA)	HepG2	Cell-SELEX	Detection	[100]
TLS11a (DNA)	HepG2	Cell-SELEX	Detection	[64]
TLS11a (DNA)	HepG2	Dual recognition and signal amplification	Cytosensor	[67]
TLS11a (DNA)	HepG2	Voltammetric based	Cytosensor	[68]
LY-1 (DNA)	HCCLM9	Quantum dots and magnetic particles	Prognostic probe	[62]
SLeX-AP (DNA)	Circulating tumor cells	Biocompatible transparent nanostructured substrates	Controlling personalized treatment	[63]
TLS11a (DNA)	HepG2	‘Activatable’ aptamer-based fluorescence probe	Detection and imaging	[71]
Bio-TLS11a (DNA)	HepG2	Streptavidin-fluorescent silica nanoparticles combination	Detection and imaging	[70]
TLS11a (DNA)	HepG2	Aptamer- based electrochemical biosensors	Detection and imaging	[65]
TLS11a (DNA)	HepG2	Label-free microcantilever array	Detection	[66]
AS1411 (DNA)	Nucleolin	AS1411-Dox adduct	Drug delivery	[76]
TLS11a-GC (DNA)	LH86	SELEX	Drug delivery	[77]
TLS11a (DNA)	MEAR	Aptamer-biodegradable polymer	Drug delivery	[79]
EPAP (RNA)	EpCAM	Aptamer-gene therapy	Therapy	[81]
OPN-R3 (RNA)	Osteopontin	SELEX	Therapy	[74]
GT75 (DNA)	Elongation factor 1A	Liposome-aptamer	Drug delivery	[78]
AFP (RNA)	Alpha-fetoprotein	SELEX	Detection and therapy	[83]
Ep-MNPs (DNA)	EpCAM	Magnetic nanoparticle-aptamer	Imaging and therapy	[86]
AP273	Alpha-fetoprotein	CE-SELEX	Imaging and therapy	[84]

### Aptamer for HCC diagnosis

The starting point of aptamer research in HCC was the development of a diagnostic tool. By using an aptamer generated by a cell-SELEX method, whole live HCC cells can be recognized. Aptamer that specifically binds to HCC cells in tissue samples and cell lines may facilitate the discovery of novel biomarker and ideal nanoparticle for HCC early diagnosis. TSL11a is one of the most studied aptamers, also in combination with other molecular probes to improve performance [64]. The TLS11a-based electrochemical biosensor (aptasensor) had been proposed as a simple, selective, and label-free diagnostic tool for the detection of the HepG2 cells. This conjugated aptamer could detect cancer cells at a very low concentration (2 cells/mL) with a wide linear dynamic range [65]. The TLS11a aptamer-based microcantilever biosensor with similar fundamental principle and function could also detect HepG2 cells, even though its sensitivity was less than the previous method (300 cells/mL) [66]. Another type of aptamer diagnostic tool using biosensor technology is a cytosensor aptamer that was developed based on a dual recognition and signals amplification strategy. In this method, TLS11a was covalently conjugated to a gold nanoparticle and horseradish peroxidase (HRP) for a sensitive detection of 30 cells/mL [67]. In a sequent development, this aptamer together with an indium tin oxide electrode assay and multifunctional nanoprobes improved the detection limit to 10 cells/mL [68]. These cytosensors have a great potential for the development of HCC diagnostic tools.

In recent years, fluorescent silica nanoparticles have been successfully used in cancer imaging due to its photostability, brightness, and high emission [69]. Biotin-labelled TLS11a combined with streptavidin-modified fluorescent silica nanoparticles showed promising results. This nanoparticle has no significant toxicity effects, both *in vitro* and *in vivo*, with stronger and more photo-stable fluorescence signal compared to conventional fluorescein isothiocyanate (FITC)-labelled aptamer [70].

The latest diagnostic approach using TLS11a aptamer is by conjugating the aptamer with fluorescence probe that can distinguish the presence of cancer and non-cancer cells. By using HCC cell lines and frozen HCC tissue section, the probe emitted a strong fluorescent signal only in the presence of the target cancer cells. This versatile technology can be adapted for other aptamer sequences targeting various cancers and diseases [71].

In a recent study, the aptamer-based chip had been developed to detect the circulating tumor cells (CTC) in blood samples of HCC patients. The SLex (aptamer for carbohydrate sialyl Lewis X) coated onto hydroxyapatite/chitosan nanofilm aptamer had clinically potential in detecting the CTCs, both for HepG2 in artificial blood and more importantly for blood from HCC patients. The detection of CTCs by using this system was significantly correlated with the tumor size, portal vein tumor thrombus, and the tumor-node-metastasis stage [63].

### Aptamers for HCC-targeted therapy

HCC-targeted therapy is one of the most promising applications for aptamers. Aptamers can be utilized as a direct therapeutic agent and as a nano-delivery agent by conjugating the aptamer with an anti-cancer drug, nanoparticles, and gene therapy [72, 73]. A new RNA aptamer targeting osteopontin (OPN-R3) showed a good efficacy in down-regulating the epithelial-mesenchymal transition and the growth of HCC in a mouse model. Mouse injected with this aptamer showed significantly decreased tumor burden compared to control and mutant control aptamer group in *in-vivo* bioluminescence imaging [74]. This study was significant in the “proof-of-concept” study in breast cancer cells that this aptamer was relevance for modifying tumor growth and metastasis [75].

A conjugation between the aptamer and anti-cancer drug demonstrated that the aptamer was able to deliver the anticancer drug precisely into the target cell or tissue. Doxorubicin-conjugated AS1411 (AS1411-Dox) had been proposed to be a simple technique to form Drug-DNA Adduct in *in vitro* and *in vivo* models of HCC. In tissue staining, the aptamer could clearly differentiate the HCC tissue and non-HCC tissue. AS1411-aptamer were specifically bound in tumor regions compared with adjacent non-tumor tissue. Moreover, AS1411-aptamer showed the strongest staining in the most abundant area of nucleolin [76]. Doxorubicin was also conjugated with the TLS11a-GC aptamer, which specifically targeted LH86 HCC cells [77].

Conjugating an aptamer with other nanoparticles will increase effectiveness. Scaggiante *et al.* generated an aptamer-liposome intercalation that increases the bioavailability of the aptamer in targeting the elongation factor 1A (eEF1A) in an HCC model. The synergic effect of the aptamer-liposome and either bortezomib or idarubicin impaired the vitality of the HCC cells in a dose- and time-dependent manner [78]. Conjugation between TLS11a with a biodegradable polymer nanoparticle effectively bound to HCC cells. It showed a higher therapeutic effect when loaded with doxorubicin compared with nanoparticle without the targeting aptamer [79]. Another study in HCC showed that nanoparticles contained peptide-modified aptamer (ST21) significantly increase the cellular uptake *in vitro* and therapeutic efficacy in mouse *in vivo* model indicating a highly efficient co-delivery vehicle for tumor-specific therapy [80]. Collectively all this work indicated the advantages of aptamers as chemotherapeutic nano-vehicle to minimize the toxicity effect and to increase the drug efficacy, at least in *in vitro*.

The use of recombinant adenovirus carrying a tumor suppressor gene into HCC is an effective gene therapy method. However, its application is still hindered by auto-immunogenicity, low stability, and non-specific toxicity to normal cells. A novel aptamer-based gene delivery system by using RNA aptamer conjugated to tumor suppressor gene can be an effective strategy. An aptamer conjugated with Ad5-PTEN had been shown to target the epithelial cell adhesion molecule *in vitro* and *in vivo*. This method significantly inhibited cell proliferation and cell migration in HepG2 with no toxic effect observed in healthy liver cells. Moreover, it induced cell apoptosis in aggressive HepG2 xenograft in nude mice without concentration-dependent toxicity [81].

### Aptamers for theranostic approach

**Figure 2:**
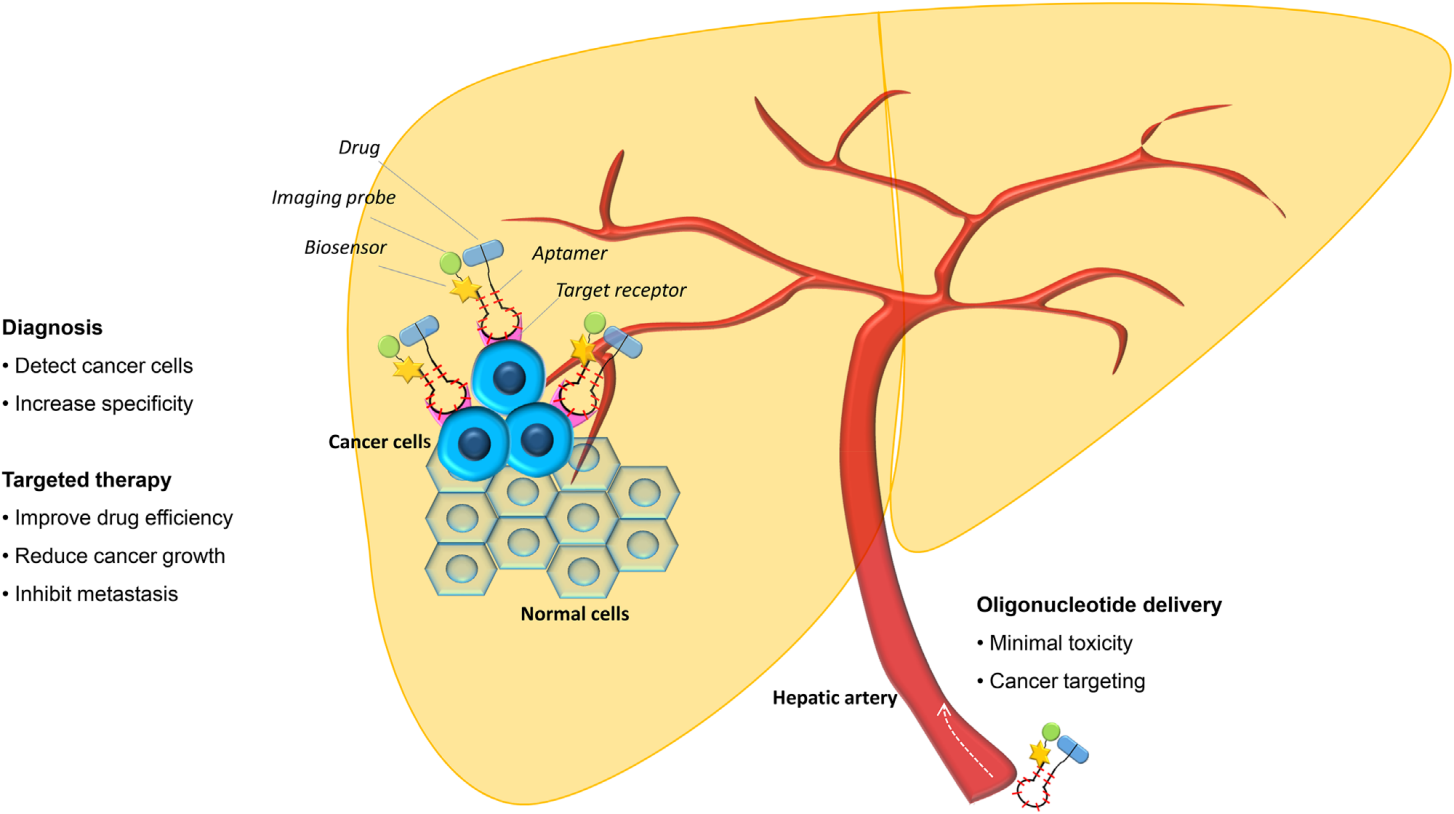
Aptamer for HCC diagnostic and targeted therapy Aptamer can act both as a diagnostic tool in the conjugation with imaging probe and biosensor and a targeted therapy agent by its neutralizing effect and by its combination with antineoplastic drugs and/or gene therapy.

Theranostic nanoplatforms (a combination of a diagnostic and therapeutic method) is a promising approach to overcome the limitation of conventional cancer therapy and diagnosis Figure 2. Because aptamers are versatile, they can be modified and improved to obtain a specific application to treat cancer. Evaluation of aptamers, both *in vitro* and *in vivo*, had considered them as a powerful theranostic agent for their potential as dual therapeutic and diagnostic application [82].

Theranostic aptamer was developed in many different ways due to its superior performance in solid tumor penetration over antibody [37]. Aptamer can be functional both as simultaneous diagnostic and therapeutic agent by combining its greater efficacy in targeting the cancer cells with its agonist/antagonist capability in cellular pathways. However, the information regarding theranostic approach against HCC is still limited. In 2012, a first report demonstrated that an RNA aptamer against alpha-fetoprotein (AFP) was able to detect cancer cells, inhibit the proliferation of AFP-associated HepG2 cells and decrease the gene expressions of the c-jun and c-fos oncogenes [83]. By using a CE-SELEX technology, aptamer AP273 against AFP had also been used to screen and identify HCC. AP273 had been found to competently modulate cancer cells by inhibiting the migration and invasion of HCC cells after *in vivo* transfection [84]. These AFP-specific aptamers could be a useful theranostic agent against AFP-related studies of HCC.

Furthermore, by integrating imaging and nano-delivery functionalities in a single agent, aptamer-based theranostic provided a novel solution for an early diagnosis that can be followed by *in situ* drugs released [85]. In 2014, a smart magnetic nanoparticle-aptamer probe targeting epithelial cell adhesion molecule (EpCAM) had been developed as a novel theranostic approach. Besides demonstrating an efficient *in vitro* magnetic resonance imaging of HCC cells, this nanoprobe also improved the delivery of the doxorubicin into the cancer cells. This nanoprobe had 98% doxorubicin entrapping efficiency and 24% doxorubicin loading efficiency which was very specific to cancer cells but not in normal cells [86].

## SUMMARY AND FUTURE PERSPECTIVE

The literature reviewed above shows promising data on oligonucleotide aptamer as a considerable potential in the development of nanomedicine against HCC. In addition to its potential *in situ*, aptamer-based targeted diagnosis and therapy can be administered in the circulation [76]. Systemic administration not only can detect the presence of the tumor cells, but it can also exhibit potent antitumor activity and may reduce tumor metastases with limited or even no side effect [87]. Nevertheless, this technology is still relatively novel and still faces several challenges to achieve its final target in a clinical setting. Recently, only a few aptamers have reached clinical trials, and there is still no approved aptamer for HCC diagnosis and therapy. Furthermore, HCC is a highly heterogeneous disease with distinct molecular profiles related to different etiologies, subtypes, and long-term development. The main challenges of aptamer developments as a nanomedicine against HCC are represented by the selection of the efficient and specific molecular target, the chemical and biological activity of aptamers *in vivo*, and by the improvement of delivery method to obtain potent aptamers. It is understood that the process of aptamer selection is lengthy and the validation will be needed in different sets of samples and models. Therefore, a target molecule must be carefully selected for a specific and effective approach.

In summary, oligonucleotide aptamer is an emerging and promising nanomedicine for HCC diagnosis and therapy in the future. Aptamer can be a powerful tool with unique and distinctive characteristics that will give positive impacts, both in basic research and clinical application of HCC. However, translating the potential of oligonucleotide aptamer from pre-clinical study to clinical application is still challenging. Many aptamers with potent functions are susceptible to endogenous nuclease degradation and have short half-lives in a biological system. Nevertheless, current and upcoming technologies on aptamer modification and stabilization by using modified nucleic acids and chemical agents will enhance the function and the stability of the aptamer for clinical use [88, 89].

We predict that in a near future aptamer technology will continue to exponentially grow and to be progressively used in the development of new efficacious aptamer-based diagnostic and therapeutic agents towards cancers, including HCC. Further developments would be needed to facilitate clinical translation of the promising preclinical studies in both HCC diagnosis and targeted therapy.
