# Correction: Aptamer: A potential oligonucleotide nanomedicine in the diagnosis and treatment of hepatocellular carcinoma

**DOI:** 10.18632/oncotarget.24572

**Published:** 2018-02-26

**Authors:** Rusdina Bte Ladju, Devis Pascut, Muhammad Nasrum Massi, Claudio Tiribelli, Caecilia H.C. Sukowati

**Affiliations:** ^1^ Fondazione Italiana Fegato, AREA Science Park Basovizza, Trieste, Italy; ^2^ Faculty of Medicine, Hasanuddin University, Makassar, Indonesia

**This article has been corrected:** The updated Acknowledgments information is provided below:

**ACKNOWLEDGMENTS**

RBL is supported by fellowships from the Lembaga Pengelola Dana Pendidikan (LPDP) of the Indonesian Ministry of Monetary, DP by NECTE project of PAR-FSC_2007-13 decree n° 3984/LAVFOR.ISTR/2014 Friuli Venezia Giulia, CHCS fellowship was partially supported by grant NIH U01AA020821. This work was supported by in-house grant from the Fondazione Italiana Fegato, Trieste, Italy, and the Faculty of Medicine, Hasanuddin University, Makassar, Indonesia.

Original article: Oncotarget. 2018; 9:2951-2961. https://doi.org/10.18632/oncotarget.23359

